# The ‘Saw but Forgot’ error: A role for short-term memory failures in understanding junction crashes?

**DOI:** 10.1371/journal.pone.0222905

**Published:** 2019-09-23

**Authors:** Chloe J. Robbins, Harriet A. Allen, Karl A. Miller, Peter Chapman

**Affiliations:** School of Psychology, University of Nottingham, Nottingham, England, United Kingdom; Tongii University, CHINA

## Abstract

Motorcyclists are involved in an exceptionally high number of crashes for the distance they travel, with one of the most common incidents being where another road user pulls out into the path of an oncoming motorcycle frequently resulting in a fatal collision. These instances have previously been interpreted as failures of visual attention, sometimes termed ‘Look but Fail to See’ (LBFTS) crashes, and interventions have focused on improving drivers’ visual scanning and motorcycles’ visibility. Here we show from a series of three experiments in a high-fidelity driving simulator, that when drivers’ visual attention towards and memory for approaching vehicles is experimentally tested, drivers fail to report approaching motorcycles on between 13% and 18% of occasions. This happens even when the driver is pulling out into a safety-critical gap in front of the motorcycle, and often happens despite the driver having directly fixated on the oncoming vehicle. These failures in reporting a critical vehicle were not associated with how long the driver looked at the vehicle for, but were associated with drivers’ subsequent visual search and the time that elapsed between fixating on the oncoming vehicle and pulling out of the junction. Here, we raise the possibility that interference in short-term memory might prevent drivers holding important visual information during these complex manoeuvres. This explanation suggests that some junction crashes on real roads that have been attributed to LBFTS errors may have been misclassified and might instead be the result of ‘Saw but Forgot’ (SBF) errors. We provide a framework for understanding the role of short-term memory in such situations, the Perceive Retain Choose (PRC) model, as well as novel predictions and proposals for practical interventions that may prevent this type of crash in the future.

## Introduction

An in-depth study of motorcycle crashes in the UK [1], revealed that more than 25% of fatal crashes involving a motorcyclist involved another road user moving into the path of the motorcyclist, typically at a junction. This corresponds to approximately 90 deaths in the UK per annum [2]. In the US, there were 5,172 motorcyclists killed on the roads in 2017 [3], and previous estimates have suggested that around 50% of US motorcycle crashes may involve other vehicles pulling into the motorcyclist’s path [4]. European analyses have suggested that in 63% of crashes involving a powered two-wheeler and another vehicle, a traffic scanning error on the part of the other vehicle’s driver contributed to the crash [5]. Based on international crash statistics [6], it is possible to estimate that around 100,000 fatalities per year may come from this type of crash. Clearly any research that improves our understanding of these crashes, and the kind of countermeasures that can be used to prevent them, has the potential to be a major contribution to world health.

Typical interpretations [7] of these junction crashes are based on the idea that the driver pulling out of the junction has failed to devote sufficient attention [8] to the traffic on the road he or she is entering, thus, they are often termed ‘Look but Fail to See’ (LBFTS). It is proposed that the crash is caused by failing to spot an oncoming vehicle. This is consistent with the psychological phenomena of change blindness and inattentional blindness, with explanations suggesting that even when attention is on an object it is not always associated with the detection and processing of this object [9, 10].

Other previous research has suggested that motorcycle accident risk is inflated due to the size-arrival effect, which suggests that smaller objects are perceived as further away, and to arrive later than larger objects [11]. Due to this perceptual error, drivers may adopt a smaller gap at the junction when a motorcycle is approaching compared to a larger vehicle such as a car or large goods vehicle. Data from both real and experimental simulations have found that crashes do occur when a car pulls into the path of an oncoming motorcycle, with the car driver thinking the motorcycle is further away than it actually is [11, 12].

In light of this, the initial intention of the current series of studies was to explore whether drivers show systematic biases in attention towards, and memory detail for different vehicle types. We analysed drivers’ eye movements and tested their ability to report vehicles approaching junctions in a high-fidelity driving simulator. Although subtle biases in memory for vehicle locations were found, the most striking finding from the first study was not the subtle biases in memory detail, but the complete failure to report some vehicles, particularly approaching motorcycles.

Short-term memory is responsible for the encoding, temporary storage and retrieval of information for complex cognitive tasks [13] and thus, this system is responsible for offering drivers feedback about the traffic situation a few seconds earlier [14]. Therefore, drivers’ ability to report vehicles depends not only on them being successfully encoded, but also on the storage of this information and its retrieval from short-term memory [15]. This suggests that report failures might not always be due to a failure in visual attention (encoding), which many previous researchers have suggested [8], but could sometimes be due to subsequent failures in memory [16].

Theoretically this should not be surprising as it has previously been argued that attention and memory modulate the comprehension of events [17, 18]. Memory errors are also a plausible explanation for driving errors, as previous studies which have investigated drivers’ memories for vehicles on the road [19–21] have found that drivers’ memories for their current environments are relatively poor [20]. Two further studies were thus conducted, using similar scenarios to the first, to explore the possible reasons for a complete failure to report approaching vehicles at junctions. We wanted to decide whether approaching vehicles were attended to on occasions where drivers failed to report them, and in particular, on occasions where drivers were willing to complete a manoeuvre in front of these oncoming vehicles.

The second and third studies analysed drivers’ eye movements, and their ability to report oncoming vehicles and their locations, at the time drivers were making a risky decision to pull out in front of approaching vehicles. In the second study we again found that there were occasions where drivers completely failed to report approaching vehicles, particularly motorcycles. Given that fixations on objects have been seen to be the best predictor of memory [22], it was important to investigate drivers’ eye movements in more detail. The third study additionally investigated drivers’ subsequent visual search after fixating on the motorcycle, on occasions where the driver failed to report it. Distinguishing between perceptual and memory explanations is a difficult task. However, post-event processes, i.e. ones that occur after the vehicle has been presented, should not influence the initial attention to and encoding of vehicles. Analysis of eye movements after fixating an oncoming motorcyclist allows us to explore the possibility that new information can interfere with the storage and retrieval of previous information held in short term memory [23].

To anticipate our results, failures to report a motorcycle were not predicted by how long a driver fixated on the vehicle, but were associated with their subsequent behaviour i.e. drivers were more likely to forget an oncoming motorcycle if they had made several head movements between looking at it and the subsequent memory test.

The raw data for the three studies presented in this paper can be found at: Robbins, C. J., Allen, H. A., Miller, K., & Chapman, P. F. (2019, May 10). The “Saw But Forgot” error. Retrieved from osf.io/nd6ug

## Study 1: Drivers’ behaviour and recall at junctions

The main aim of Study 1 was to investigate drivers’ visual attention towards, and memory for the location of vehicles at junctions. We expected that drivers would have reduced attention towards smaller vehicles, as well as estimating smaller vehicles to be further away than larger vehicles. Given that this experimental set up requires drivers’ memory for vehicles and their locations to be tested on numerous occasions, this study also tested whether the presence of a memory test changed participants’ behaviour.

### Participants

All studies had full ethical approval from the University of Nottingham Psychology ethics committee. All methods were carried out in accordance with the relevant guidelines and regulations, with written informed consent obtained by all participants.

The sample size for Study 1 was determined a priori based on a power analysis (medium effect size for repeated measure, within factor difference, Cohen’s f = 0.25, 1-β = 0.90, *p* = .05) [24]. This was because our main comparisons of interest were the within groups differences in reporting location of approaching vehicles.

#### Drive only group participants

Data were collected from 30 participants (Mean age = 21.8, SD = 4.5, Range = 18–40; Male = 12, Female = 18) who had held a driving licence for between 1–22 years. They had a reported annual mileage between 0–10,000 miles (Mean = 3,670) and a total mileage between 30–120,000 miles (Mean = 21,826). Eight of the participants were recruited for first year undergraduate credit, six participants were recruited as part of a study swap and sixteen of the participants received a £5 inconvenience allowance for their time.

#### Memory test group participants

Data were collected from an additional 30 participants (Mean age = 21.2yrs, SD = 2.9, Range = 18–31; Male = 23, Female = 7) who had held a driving licence for between 8 months- 9 years. They had a reported annual mileage between 0–10,000 miles (Mean = 4,140) and a total mileage between 0–50,000 miles (Mean = 8,581). All participants received a £5 inconvenience allowance for their time.

### Design

All driving scenarios required the participant to drive up to the same intersection. The intersection was a cross-road, therefore traffic could theoretically be coming from the left, right or straight ahead, although in the key trials in the current studies the road straight ahead on the other side of the junction was always clear. The driver did not have right of way over vehicles approaching from the left or right on the main road. The driver completed the scenario by pulling out of the junction when it was deemed to be safe and continuing straight on down the minor road at the far side of the junction.

A 2x3x3 mixed design formed the core of the study, with the between groups factor referring to whether the driver received memory tests (Memory Test Group vs. Drive Only Group). One within groups factor was the type of oncoming vehicles at the junction. On key trials there were always two oncoming vehicles, one from the left and one from the right. One of each pair was always a car, while the second varied such that there were three possible combinations (car-car, car-motorcycle, car-large vehicle). The vehicles on the two sides of the junction always came from the same distance as each other, and this distance formed the second within groups factor with three levels, (far distance (95m), medium distance (60m), near distance (25m)). Both oncoming vehicles were always visible when the participant arrived at the junction and were already travelling at a fixed speed of 30 mph. Each vehicle combination was repeated twice at each distance, with left and right vehicles swapped in location, providing 18 target trials (3 x vehicle type, 3 x distance, 2 x LR vs RL). Participants encountered these 18 key target trials along with 12 general traffic trials, where traffic was randomly generated in an unpredictable manner by the simulator. This totalled 30 trials, with the order being fully counterbalanced.

#### Memory test group only

Twelve of these 30 trials were memory test trials. On these trials, the scenario was terminated at the point where the driver reached the junction and a memory test was given. Drivers had to verbally indicate what vehicles they saw at the junction and using a laser pointer, indicate the location of each oncoming vehicle. These memory test trials consisted of 9 target trials and 3 general traffic trials. The 9 target trials were three of each vehicle combination (car-car, car-motorcycle, car-large vehicle), at each distance (near, medium and far).

### Apparatus

Study 1 took place in the Nottingham Integrated Transport and Environment Simulation (NITES) facility’s, high fidelity driving simulator (NITES 1). This simulator comprises of a full BMW Mini, housed within a projection dome and mounted on a six-degrees of freedom motion platform with a 360-degree projection screen. The scenarios were formed on the screens using six projectors.

XPI (XPI Simulation, London, UK) driving simulation software was used to create the scenarios. All scenarios took place at the same cross road intersection, which was based on an urban road. The intersection had a “Stop sign” at the end, reminding participants to check the junction before pulling out. The junction chosen for the scenarios was a flat junction, with houses either side of the road on the approach. The junction had equal visibility to the left and right when participants stopped at the junction. Each scenario started in the same location, which was around 80m from the junction. The scenario ended just after the participant had pulled out of the junction and continued straight on at the junction for around 30m. The speed of the approaching target vehicles remained constant throughout the experiment, travelling at 30mph, chosen as this is the average speed of both cars and motorcycles on British roads [25].

Drivers’ eye movements were tracked using two linked FaceLAB 5.0 remote eye tracking systems (four cameras and two infrared sources), which allowed participants’ eye movements to be tracked continuously over a range of approximately 120 degrees in front of the driver.

A KODAK PIXPRO 360-degree action video camera was mounted on top of the BMW Mini roof, directly above the driver’s head, and not visible to the driver. This camera allowed for the full 180-degree front field of view to be visible. In the memory test condition these recordings were used to measure the location of the two oncoming vehicles at the time the simulation was paused. These locations were measured relative to the front of the vehicle (closest point to the driver) allowing us to calculate the visual angle away from straight ahead at the moment the simulation stopped. This measure was compared to the estimated locations of the vehicles as indicated by the participant shining a laser pointer onto the blank simulation screens and recorded using the same video camera.

### Procedure

Participants completed a short ‘Driving Experience’ questionnaire which included questions on age, gender, years of licensure as well as miles and hours driven. The driver entered the driving simulator and the eye trackers were calibrated. Participants drove up to a series of intersections where there was oncoming traffic that had the right of way. They were instructed to approach the junction at a speed of around 20mph and drive across the junction, choosing to go either before or after the oncoming traffic arrived. They were encouraged to drive as naturally as possible throughout the experiment and obey all road signs. The first two trials served as practice trials, allowing participants to become familiar with the simulator as well as checking for any signs of simulator sickness. On average, each scenario took around 20 seconds to complete.

For drivers in the Memory Test condition, drivers were informed that on selected trials, the scenario would be terminated when they reached the junction, leaving a white simulator screen. On these trials, the participant should indicate verbally what vehicles they saw at the junction and using the laser pointer, indicate the location of each oncoming vehicle. Participants could not predict on which trials the memory test would occur.

### Results

#### Dependent measures

For behavioural and eye movements measures, the dependent variables included Approach Time [26], Number of Stops, Wait Time and Cross Time [27], as well as Mean Fixation Duration, Proportion of Fixations and Proportion of Gaze [26]. A custom MatLab [28] script was used to automatically analyse these behavioural and eye movements.

Specifically, the MatLab script obtained the measure of Approach Time by calculating how long it took drivers to travel through the ‘approach zone’, which started at 35m away from the junction and finished at the moment where the front of the driver’s car had entered the junction by crossing the junction entry line. The ‘approach zone’ thus started when the target vehicles first become visible, therefore this was the point where approaching traffic may start to alter the approach behaviour of the driver. Number of Stops and Wait Time were also calculated in the ‘approach zone’. Number of Stops was calculated by the number of times the participants’ vehicle speed went below 1mph, and Wait Time was the time that passed while the participants vehicle speed remained under 1mph. Finally, Cross Time refers to the amount of time it took the driver’s car from entering the junction (crossed the give way line) until when the rear of the driver’s car had cleared the junction (crossed the junction exit line).

In regards to eye movements, due to the fact drivers could generally only look at one vehicle at a time, measures of behaviour towards the two vehicles approaching the junction were not independent of each other. For this reason, in each scenario one vehicle was always designated as the target vehicle, where the visual attention towards that specific vehicle was analysed. For the car-car trials, the red car was always the target vehicle, and in the remaining trials, the motorcycle or the large vehicle was always the target vehicle.

In addition, given that drivers made large rapid head movements and fixations at wide eccentricities, it was difficult to always be sure of the quality of the eye tracking. For this reason, a conservative approach was adopted, focussing on the broad direction of fixation (towards or away from the target vehicle, rather than requiring an unambiguous fixation on the vehicle). A fixation dispersion threshold of 0.2 of a radian for 100ms was used to regard a fixation to be in progress. This wide threshold meant that smooth pursuit movements to oncoming vehicles were treated as equivalent to fixations. Proportion of Fixations was calculated by measuring the number of fixations towards and away from the target vehicle side of the junction. The proportion of all these fixations towards the target vehicle side of the junction was then calculated. The Proportion of Gaze was calculated in the same way as the previous measure with total gaze duration rather than number of fixations. Total gaze duration was the total time spent on fixations to the target vehicle side of the junction, so Proportion of Gaze gives a general measure of how much visual attention was towards the oncoming target vehicle. Mean Fixation Duration was calculated by dividing the number of fixations made towards the target vehicle side of the junction, by the total gaze duration towards the target vehicle side of the junction.

Drivers’ reports of vehicles were coded as a dichotomous variable i.e. whether drivers ever failed to report a vehicle or notoss Time et v proproation of gaze on the target v ehciles. in groups differences in rerporting y on h the g. eaving a white, and therefore Cochran Q tests were conducted to investigate this measure as a function of Vehicle Type and Vehicle Distance. This simple coding of the variable was chosen as the frequency of forgetting was not high enough to justify another test. Finally, for drivers’ recall of vehicle locations, these were analysed by Driver Bias. Driver Bias was defined as the difference between the actual visual angle of the vehicle from straight ahead at the moment the simulation stopped, and the estimated location of the vehicle as indicated by the participant’s laser pointer. This value could have been negative (an overestimation of the visual angle with it being reported as further away than it really was) or positive (an underestimation of the visual angle with it being reported closer than it really was).

#### Effect of memory test on driving behaviour

There was a difference in driving experience between the Memory Test Group and Drive Only Group. To assess the effect of this confound, a correlation between experience and the key behavioural and eye movement measures was conducted (S1 Table). No significant correlations were found.

To test if the memory testing changed driver behaviour, a behavioural and eye movement comparison between the Memory Test Group and the Drive Only Group was conducted using a 2x3x3 mixed ANOVA, with a between group factor of Group (Memory Test or Drive Only) and within group factors of Vehicle Type (Car, Motorcycle, Large Vehicle) and Vehicle Distance (Near, Medium, Far).

There were no significant differences between groups in Approach Time [F (1, 58) = 2.58, MSe = 9.51, *p* = .11], Number of Stops [F (1, 58) = 7.15, MSe = .38, *p* = .61], Wait Time [F (1, 58) = 1.76, MSe = 157.55, *p* = .19] and Cross Time [F (1, 58) = .19, MSe = 1.78, *p* = .67]. There was also no difference in drivers’ Proportion of Fixations [F (1, 58) = 2.49, MSe = .24, *p* = .12] and Proportion of Gaze [F (1, 58) = 3.78, MSe = .23, *p* = .07] however, drivers’ Mean Fixation Durations were significantly longer in the Memory Test Group compared to the Drive Only Group (F (1, 58) = 8.91, MSe = 288575.98, *p* < .01). See S2 Table for full descriptive statistics for both groups.

#### Drivers’ memory for vehicles and locations

**Fig 1:**
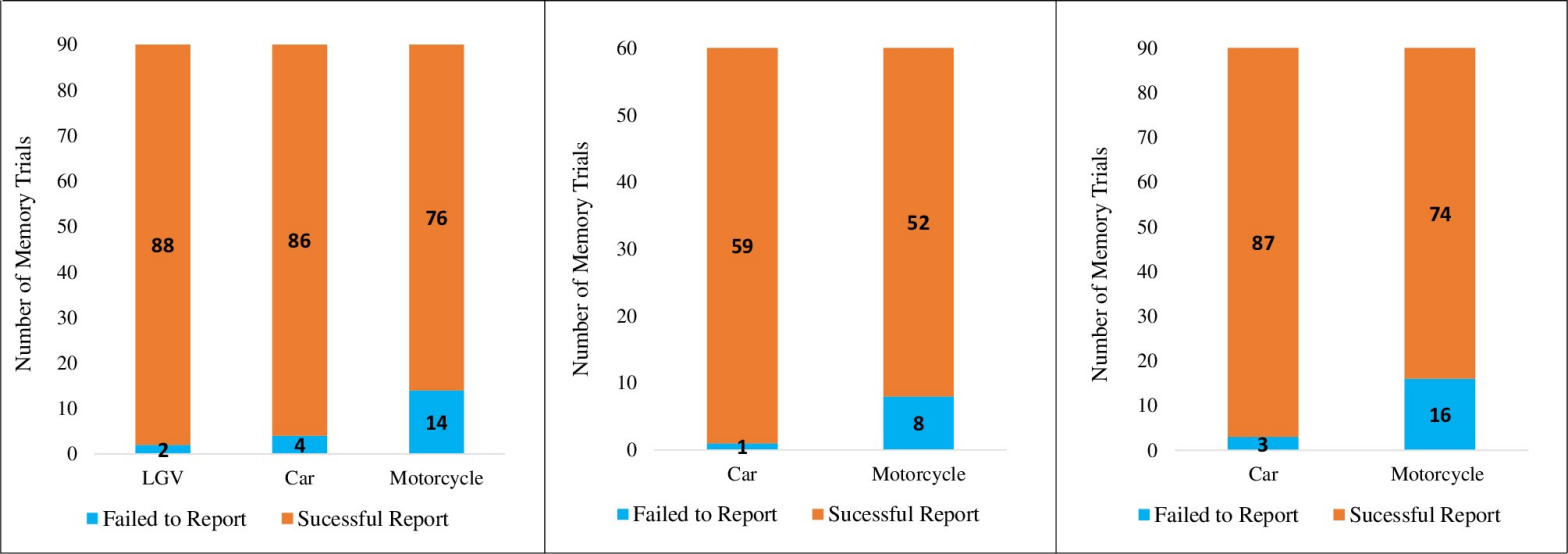
The number of occasions drivers failed to report a vehicle and successfully reported a vehicle (A) in Study 1, (B) in Study 2 and (C) in Study 3.

The results from the memory test trials were analysed. Although it was expected that drivers would report all approaching vehicles, they failed to report one of the two oncoming vehicles on 7.4% of trials (20 occasions). These were split between failures of memory for motorcycles (14) cars (4) and LGVs (2), (Fig 1A). There was a difference in failure to report a vehicle as a function of Vehicle Type *(χ*^*2*^*(2) = 17*.*73*, *p <* .*001)*, showing that drivers failed to report significantly more motorcycles than cars and large vehicles *(p <* .*001)*. There was also a significant difference in drivers’ reports of vehicles as function of Vehicle Distance *(p <* .*01)*, indicating that drivers failed to report more far target vehicles compared to medium and near distance vehicles.

For the location of vehicles, there was an overall tendency for drivers to underestimate the visual angle of oncoming vehicles, with drivers remembering oncoming vehicles to be closer than they really were (mean difference between true angle and indicated location = 8.7 degrees). There was a significant main effect of Vehicle Type (F (2, 58) = 5.71, MSe = 46.24, *p* < .01), with this tendency being stronger for motorcycles (11.72°) than for cars (8.48°) and LGV (5.79°) (p<0.01). There was also a main effect of Vehicle Distance (F (2, 58) = 11.23, MSe = 55.68, *p* < .001), with this tendency being more pronounced for far vehicles than for medium or near vehicles (p < .001).

#### Eye movements during memory testing

Given the surprisingly frequent memory failures for motorcycles in the memory test group, we conducted additional detailed analysis of eye movements for these participants on the trials where their driving was not interrupted by a memory test. For this group, drivers’ eye movements in the direction of the target vehicle were analysed using a series of 3x3 within subject ANOVAs, with factors of Vehicle Type (Car, Motorcycle, Large Vehicle) and Vehicle Distance (Near, Medium, Far). For the factor of Vehicle Type, two a priori orthogonal contrasts were specified that first compared motorcycles with cars and large vehicles together and secondly compared cars with large vehicles. For the factor of Vehicle Distance, contrasts were specified which tested for linear trends in the data.

Drivers had lower mean fixation durations to the side of the junction where a motorcycle was present (337.67 ms) compared with cars and large vehicles (415.68 ms) (Main effect: F (2, 58) = 11.75, MSe = 81303.89, *p* < .001; contrast for motorcycles vs. cars and large vehicles: F (1, 29) = 4.21, MSe = 130135.62, *p* = . 05). However, there was no main effect of Vehicle Type for either Proportion of Fixations (F (2, 58) = 1.54, MSe = .08, *p* = .22) or Proportion of Gaze (F (2,58) = 2.01, MSe = .11, *p* = .14). These findings were consistent with the same analysis conducted for the Drive only and Memory test groups–S1 File. Thus although mean fixation durations were slightly lower, there was no evidence that drivers were looking at motorcycles less often than cars and LGVs, or for less time overall.

### Discussion of Study 1

We had set out to investigate drivers’ visual attention towards, and memory for different vehicle types approaching a junction. Firstly, we can conclude that drivers generally did not change their behaviour due to the presence of a memory test though they may have slightly increased their attention to oncoming vehicles as evidenced by the increase in fixation durations.

The most unexpected finding from Study 1 was that on some occasions, participants were entirely unable to report the presence of one of the oncoming vehicles, with 70% of these occasions involving oncoming motorcycles. The fact participants were unable to report oncoming motorcycles is remarkably consistent with the incidence of junction crashes involving cars pulling into the path of oncoming motorcycles, as well as predictions from inattentional blindness studies [9]. This study is therefore a novel demonstration of drivers experiencing complete report failures at a fully simulated junction, rather than just making judgments of safety about still images [8].

Although we found an overall significant underestimation of vehicle location, with this being particularly pronounced for motorcycles, much of this effect came from trials where the driver may never have intended to pull out at the junction. This is a limitation of Study 1, as there is no measurement of whether the driver would actually have pulled out into the junction on the memory trials. Given that inattentional blindness and change blindness paradigms are based around the fact that it is expected that items are seen due to their behavioural relevance to the task [29], it is important that the vehicles in this paradigm are critical for a driver’s decision to make a manoeuvre, and not occasions where the driver is always waiting for the vehicles to pass before pulling out. The next study we report was designed to investigate the possibility of complete report failures on occasions where drivers were actually pulling out into the junction with oncoming vehicles nearby.

## Study 2: Failure to report vehicles when pulling out at a junction

This study is designed to investigate drivers’ attention towards and memory for approaching vehicles in more safety critical situations, by presenting vehicles at driver’s individual gap acceptance threshold. Gap acceptance methods are thought to produce rich sources of data, collecting data from gaps which are both accepted and rejected by drivers, and most importantly estimating the point of the ‘critical gap’ [30]. Given that the vehicles in these situations are more task relevant, with drivers having to make to more informed decision about when to pull out of the junction, the main aim is to explore whether the findings from Study 1 can be replicated on occasions where drivers are intending to pull out of the junction in front of the approaching vehicles.

### Participants

The sample size for Study 2 was determined a priori based on a power analysis. The overall sample size of 30 had more than adequate power (.95) to detect a large within subject effect (d = 0.8) with the alpha level used for analysis being *p* < .05. Data were collected from 30 drivers (Mean age = 26.4, SD = 8.7, Range = 20–59; Male = 10, Female = 20) who had held a driving licence for between 3 months- 35 years. They had a reported annual mileage between 0–15,000 miles (Mean = 4739) and a total mileage between 40–200,000miles (Mean = 53,049). All participants received a £5 inconvenience allowance for their time.

### Design

We used a recently developed paradigm that facilitates the measurement of performance when drivers pull out in front of approaching vehicles, by estimating each individual driver’s gap acceptance threshold [27]. A series of 12 trials were presented to participants, where the vehicles were approaching at fixed distances (45, 55m, 65m, 75m, 85m and 95m). There were two vehicle combinations (car-car, car-motorcycle), where one vehicle approached from the left and one approached from the right. The order of the vehicle combinations, and the direction of the approaching vehicles (left or right) was randomised at each distance. Drivers could decide whether or not to pull out in front of the approaching vehicles. These fixed distances were used to estimate the distance oncoming vehicles had to be from the junction to create a 50% chance of each individual driver accepting the gap [31, 32]. The estimate of drivers’ gap acceptance threshold could range from 40m to 100m from the junction (1m intervals). Although a gap acceptance threshold was calculated for car-car trials and car-motorcycle trials separately, there was also a combined threshold based on all trials irrespective of oncoming vehicle type.

Once the gap acceptance threshold had been calculated, approximately eight subsequent trials were conducted with the vehicles approaching from that individual’s combined threshold distance. Participants were given a memory test on the first four occasions where they began to pull out in front of these oncoming vehicles. No memory test was given on the (approximately four) occasions were the driver was not willing to pull out. The experiment continued until four memory test trials had been conducted, consisting of 2 car-car trials and 2 car-motorcycle trials.

### Apparatus

The apparatus used for the study was identical to that used for Memory Test Group in Study 1.

### Procedure

The first part of the procedure was identical to the Memory Test Group in Study 1 however, the trials the participants were exposed to differed. The participant first completed a series of fixed distance trials to estimate their gap acceptance threshold. From this, the driver’s individual combined threshold (using data from both car-car and car- motorcycle trials) was calculated and used for future trials. Drivers were then given a series of trials at this threshold, and on occasions when they started to pull out in front of oncoming vehicles they were given a memory test. On the memory test trials, the simulation was stopped at the moment the driver started to cross the junction ahead of oncoming vehicles. Drivers were then asked to report whether there were any vehicles present at the junction, and indicate the estimated location of these vehicles using a laser pointer. The experiment continued until four memory tests had been given.

### Results

#### Dependent measures

Drivers’ reports of vehicles and vehicle location were calculated using the same method as Study 1. Gap acceptance thresholds were the estimated distance vehicles had to be on car-car and car-motorcycle trials for drivers to be 50% likely to pull out in front of them.

In regards to eye movements, these were calculated and compared between trials where drivers had failed to report an oncoming motorcycle and trials were they successfully reported the motorcycle. Proportion of Fixations, Proportion of Gaze and Mean Fixation Duration to the side of the junction were defined in the same way as Study 1 using a custom MatLab [28] script. However, a fixation was only counted when it was made to the side of the junction where the target vehicle was approaching and was still visible.

For the analysis, vehicle location estimates, gap acceptance thresholds and eye movements were subject to a within subject t-test, with the factor of Vehicle Type (car vs. motorcycle).

#### Drivers’ memory for vehicles and locations

As in the previous study, a Cochran Q test was conducted for the factor of Vehicle Type. We found that drivers sometimes failed completely to report one of the approaching vehicles at the junction—failing to report significantly more motorcycles (8 occasions) than cars (1 occasion) (*χ*^*2*^*(1) = 7*.*00*, *p <* .*01*, Fig 1B). When vehicles were correctly reported we found that participants generally estimated the location of vehicles to be closer to them than they actually were by, on average 18.7 degrees. In contrast to Study 1, there was a significant difference in driver bias for the location of cars and motorcycles (t (29) = 2.46, *p* < .05), with drivers having more of a bias to estimate cars to be closer to them than motorcycles (Cars: 21.1 deg, motorcycles 16.5 deg, p < .05).

#### Drivers’ gap acceptance thresholds

The distance at which the drivers were 50% likely to pull out for car-car trials (M = 84.11m, SE = 2.63m) and car-motorcycle trials (M = 85.74m, SE = 2.42m) did not differ as a function of Vehicle Type (t (29) = 1.07, *p* = .29).

#### Drivers’ eye movements

To test whether drivers attended less to a vehicle that they later failed to report, drivers’ eye movements to the side of the junction on the eight occasions were a driver failed to report a motorcycle were compared to the other trial where they had correctly reported a motorcycle at the junction. It was found that there was no significant difference in the Proportion of Fixations [t (7) = .99, p = .36], Proportion of Gaze [t (7) = 1.04, p = .33], and Mean Fixation Duration [t (7) = 2.05, p = .08] to the side of the junction of the approaching motorcycle when drivers failed to report and successfully reported the approaching motorcycle. See S3 Table for descriptive statistics of all measures.

### Discussion of Study 2

Study 2 demonstrates that drivers can fail to report approaching vehicles even when pulling out of a junction in front of them, with this being more prevalent for motorcycles than cars. This study created trials where the oncoming vehicles were as close as they could possibly be for the individual to still spontaneously choose to pull out in front of them, creating a much more realistic scenario for representing genuinely risky manoeuvres that might potentially lead to junction crashes in the real world.

In regards to drivers’ estimated vehicle locations, there was an overall significant underestimation of estimated vehicle location, with this being particularly pronounced for cars compared to motorcycles (this differed from Study 1). This finding is consistent with research into the size-arrival effect, with the size of vehicles having an impact on the predicted motion of vehicles [11]. This is also supported by additional research focusing on road markings, which has found that drivers’ perceived distance is dependent on many factors including size of the object [33, 34]. The difference in findings for Study 1 and Study 2 is thought to be due to vehicles being presented at unrealistically far and near distances in Study 1.

Given that junction manoeuvres require drivers to retain information about traffic in memory while scanning the junction, our results suggest that it could be possible that some of the vehicles which are later unreported have been attended to and processed, but have been forgotten before the decision to pull out is made. However, a limitation of Study 2 is that we cannot confidently conclude whether drivers actually fixated on the approaching vehicle before failing to report it. As previously mentioned, given that the length of a participant’s fixation has been seen to predict memory for objects [22], it is important to firstly explore whether drivers directly fixate on vehicles when distinguishing between the explanations of a failure to attend and a failure to recall. In addition, a more explicit test of memory interference would be to explore drivers’ subsequent visual behaviour after fixating on the approaching vehicle, as we may expect subsequent information after fixating on a motorcycle to interfere with previously encoded information in working memory [23].

## Study 3: Failure to report vehicles with head-mounted eye-tracking

In Study 3 we replicated Study 2 but added lightweight eye-tracking glasses to obtain highly accurate measures of fixations at extreme eccentricities (e.g. after the driver has made a head movement to the left or right). We investigated whether oncoming vehicles were directly fixated, and investigated drivers’ subsequent visual search behaviour after fixating on the target vehicles.

### Participants

Data were collected from 45 participants (Mean age = 22.9, SD = 4.9, Range = 18–41; Male = 12, Female = 33) who had held a driving licence for between 4 months- 22 years (m = 5 years). They had a reported annual mileage between 0–16,000 miles (Mean = 3246 miles) and a total mileage between 0–154,000 miles (Mean = 20,185 miles). All participants received a £5 inconvenience allowance for their time.

The sample size for Study 3 was determined a priori, based on the number of participants who failed to report a motorcycle in Study 2. As there were 8 participants who unsuccessfully reported a motorcycle in Study 2, we calculated that we would need an overall sample size large enough to provide at least 12 participants with report failures in order to detect a difference in drivers’ eye movements on successful and unsuccessful report trials (large effect size for within subject difference, Cohen’s d = 0.8, 1-β = 0.80, *p* = .05). Based on the previous study, this required a total sample size of 45 participants, with the logic that at least 12 people will fail to report a motorcycle on at least one occasion.

### Design

The design of the study was identical to Study 2.

### Apparatus

**Fig 2:**
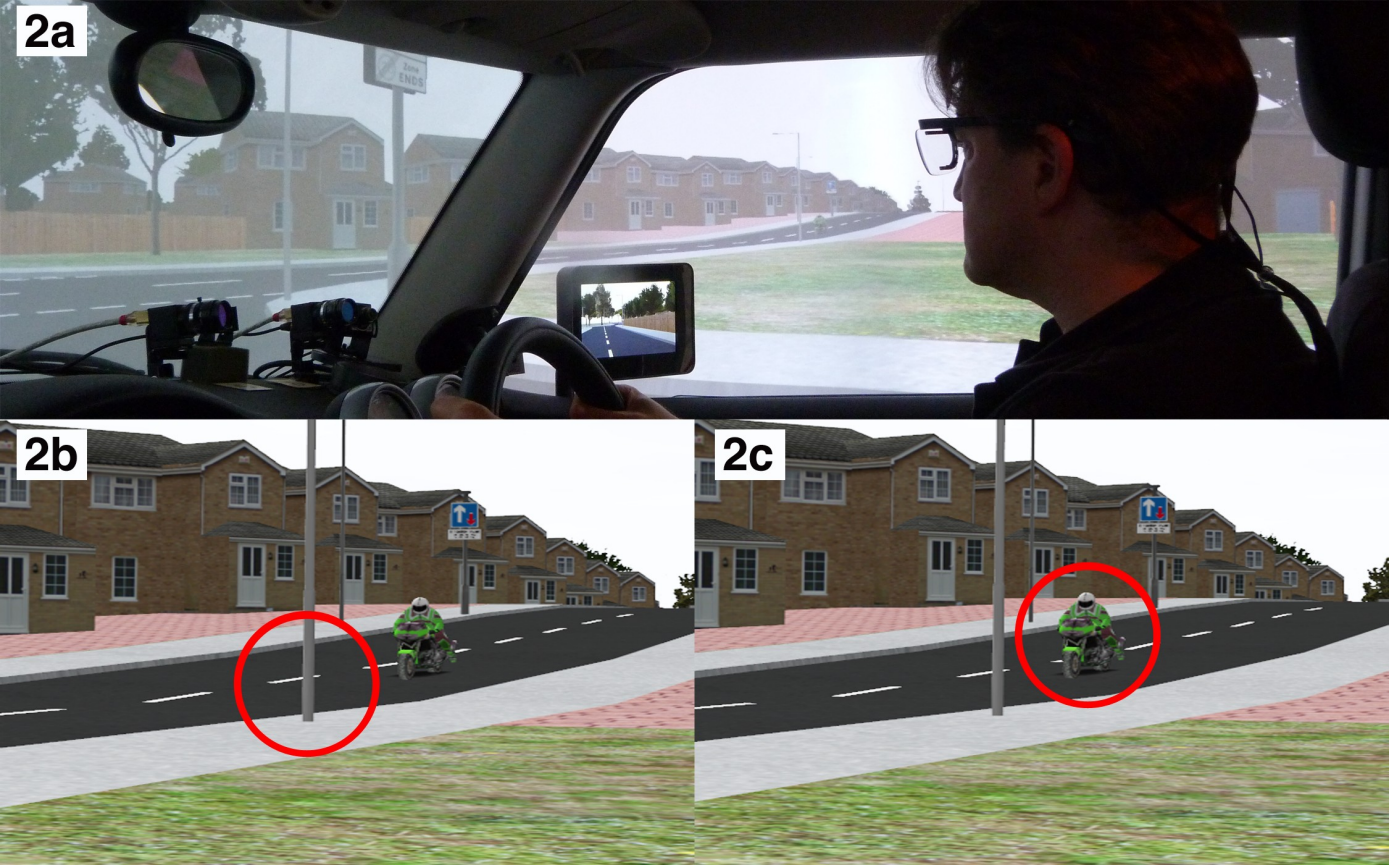
(A) A view to the right within the simulator vehicle showing one of the authors (PC) wearing the head-mounted eye-tracking glasses as used in Study 3 in a trial with a motorcycle approaching from the right-hand side of the intersection at a typical gap-acceptance threshold. Part of the FaceLab system used to record eye movements in Studies 1 and 2 are also visible on the dashboard in this Figure. (B) “Looked but Fail to See”: A close up of the view of the eye tracking on one of the five occasions when a driver failed to recall the oncoming motorcycle without having previously fixated it. The red fixation circle has a radius of 1 degree of visual angle and represents the closest fixation this driver made to the motorcycle before entering the junction. (C) “Saw but Forgot”: A close up of the view of the eye tracking on one of the eleven occasions when a driver failed to recall the oncoming motorcycle despite having previously fixated it. The red fixation circle has a radius of 1 degree of visual angle and represents an unambiguous fixation on an oncoming motorcycle that the driver will attempt to pull out in front of.

The apparatus used for Study 3 was identical to Study 2 except that eye movements were recorded using Tobii Pro Glasses 2 (See Fig 2A). These glasses were attached to a small recording unit which was kept in the car door and allowed the participant to move freely in the car, not obstructing their movement or view. The glasses tracked the participants’ pupil and corneal reflection, recording at a rate of 50hz. A successful calibration was needed for every participant before the memory trials could begin. The glasses also had a wide-angle HD scene camera in the centre (90 degrees), which captured the driver’s natural viewing behaviour. The researcher could see this live viewing of the participant’s view, overlaid with eye tracking, on a separate laptop (82 degrees horizontal, 52 degrees vertical).

### Procedure

The procedure was identical to Study 2.

### Results

#### Dependent measures

Drivers’ reports of vehicles, vehicle location and gap acceptance thresholds were calculated using the same method as Study 2.

Drivers’ eye movements were calculated and compared on trials where they failed to report a vehicle and on trials were they successfully reported the vehicle. Eye movements were manually coded using the Tobii Pro Glasses Analysis software. Drivers’ gaze on the vehicle was defined by the 1-degree radius fixation circle overlapping any part of the oncoming vehicle. When the driver’s gaze was on a motorcycle, this was manually coded as an event. When the driver’s gaze came off the motorcycle, this was coded as another event. The dependent measures on the approaching motorcycle were Number of fixations, Total Gaze Duration and Mean Fixation Duration. The Number of Fixations was the total number of times the driver’s gaze was within 0.5 of a degree for over 60ms on the target vehicle. The Total Gaze Duration was the total time spent on fixations on the target vehicle. The Mean Fixation Duration was calculated by dividing the number of fixations made on the target vehicle by the total gaze duration.

Drivers’ subsequent visual behaviour after fixating on the approaching motorcycle was also analysed, investigating the following variables: Subsequent Number of Fixations, Subsequent Number of Head Movements and Time of Last Fixation. Subsequent Number of Fixations was calculated by the number of times the driver made a fixation after the last fixation on the motorcycle until they pulled out of the junction. The Subsequent Number of Head Movements was the number of large clearly distinguishable head movements after their last fixation on the motorcycle before pulling out of the junction. Finally, the Time of Last Fixation was the time between the last fixation on the motorcycle and pulling out of the junction.

For the analysis, drivers’ vehicle location estimates, gap acceptance thresholds and eye movements were subject to a within subject t-test, with the factor of Vehicle Type (car vs. motorcycle).

#### Drivers’ memory for vehicles and locations

There were 16 drivers that failed to report a motorcycle and 3 drivers that failed to report a car when asked to recall the vehicles present (Fig 1C). There was a significant difference in drivers’ recall as a function of Vehicle Type (*χ*^*2*^*(1) = 11*.*27*, *p <* .*001)*, showing that drivers failed to report significantly more motorcycles than cars.

As previously, drivers generally underestimated the location of vehicles, estimating vehicles to be closer to them than they actually were. As in Study 2, drivers estimated cars to be closer to them than motorcycles (cars: 22.22 deg, motorcycles:15.50 deg; t (44) = 3.49, *p* < .01).

#### Drivers’ gap acceptance thresholds

The distance at which drivers were 50% likely to pull out for car-car and car-motorcycle trials did not differ as a function of Vehicle Type (Car: 82.44m; Motorcycle 81.66m, t (44) = .65, *p* = .52). A point-biserial correlation also revealed there was no correlation between drivers’ combined gap acceptance threshold and report of motorcycles, (r_pb_ = -.01, n = 45, p = .95) or cars (r_pb_ = -.15, n = 45, p = .32), indicating that drivers’ reporting of vehicles was not related to how far away they were being presented.

#### Drivers’ eye movements

On 5 of the 16 occasions on which a driver failed to report a motorcycle, the driver failed to fixate on the approaching motorcycle (Fig 2B). However, on 11 of these occasions the driver had already made an eye movement on the approaching motorcycle for at least 60ms (Fig 2C).

#### Drivers’ eye movements on the vehicle

**Fig 3:**
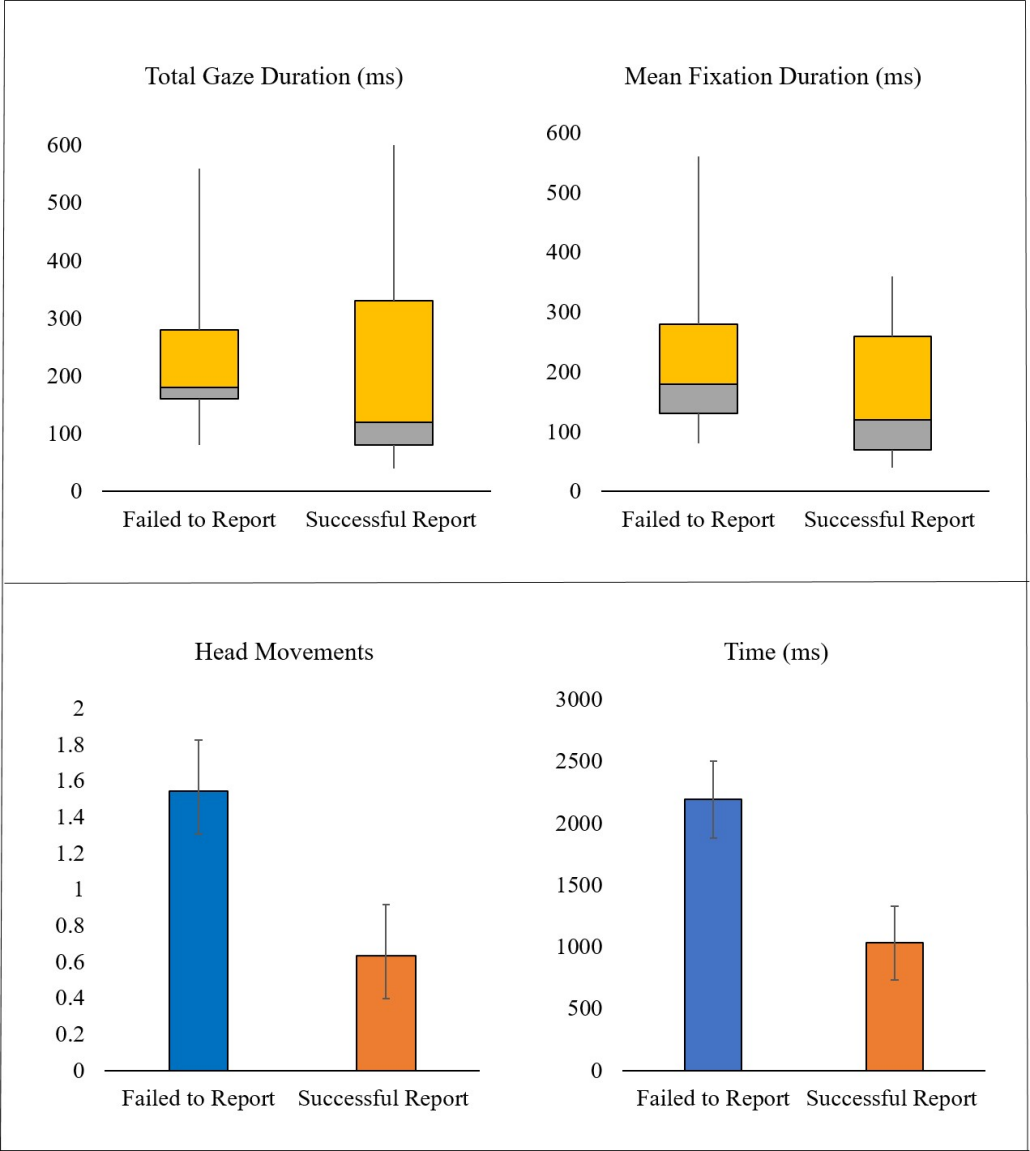
(A) Total gaze durations and mean fixation durations on reported and unreported motorcycles. (B) Post-fixation behaviour for reported and unreported motorcycles: Number of head movements and total time between fixating the motorcycle and pulling out at the junction.

For the eleven participants who fixated on the motorcycle before subsequently failing to report it, their eye movements on the occasion when they did not report the motorcycle were compared to their eye movements on a trial where they had correctly reported a motorcycle at the junction (on the left or right). There was no difference in the number of fixations on the unreported (1.09) and reported (1.36) motorcycles (t (10) = 1.40, *p* = .19) and no difference in total gaze duration or mean fixation duration on the unreported and reported motorcycles (total gaze: t (10) = .37, *p* = .72; mean fixation duration; t (10) = 1.29, *p* = .23; Fig 3A). To further clarify this result, the eye movement measures were compared between the trials where the motorcycle was not reported and matched trials where another participant reported the motorcycle. Another 11 participants who reported the motorcycle on the same side of the junction were matched on the basis of combined gap acceptance thresholds. Again, there were no differences in drivers’ eye movement measures (all p >.1), see S2 File.

#### Drivers’ subsequent eye movements after fixating the vehicle

There was no difference in drivers’ subsequent number of fixations after fixating on unreported motorcycles (3.91) and reported motorcycles (2.10) (t (10) = 1.73, *p* = .11) before pulling out of the junction. However, there was a significant difference in drivers’ subsequent number of head movements after fixating on unreported motorcycles compared to reported motorcycles (unreported: 1.55, reported: .64; t (10) = 5.59, *p* < .001) before pulling out of the junction. Drivers had more subsequent head movements when they failed to report a motorcycle compared to when they correctly reported it. There was also a significant difference in the time of last fixation on the motorcycle before pulling out of the junction, for unreported motorcycles and reported motorcycles (t (10) = 3.32, *p* < .01). The time between last fixation on the oncoming motorcycle and pulling out at the junction was more than twice as long for unreported motorcycles (2190.36 ms) compared to the reported motorcycles (1032.18 ms, Fig 3B).

In regards to cars, out of the three occasions on which a driver failed to report a car, the driver made an eye movement on the approaching car for at least 60ms on one of these occasions. Eye movements on the car, and subsequent visual behaviour after fixating on the approaching car can be seen in S3 File. Eye movement raw data and means from the 11 participants who failed to recall a motorcycle can also be seen in S4 Table.

### Discussion of Study 3

Study 3 further demonstrated that driver’s underestimation of vehicle location was more prevalent for cars compared motorcycles, which is also consistent with research into the size-arrival effect. More importantly, it was found that drivers failed to report approaching vehicles when pulling out of a junction in front of them, with this being more prevalent for approaching motorcycles than cars. These report failures were not predicted by the distances at which these vehicles were being presented, nor by how often or long the vehicles were being directly fixated. It seems that these report failures were associated with drivers’ subsequent visual search behaviour after fixating on the approaching motorcycle and before pulling out of the junction.

In particular, drivers’ subsequent number of head movements after fixating on the approaching motorcycle, as well as the time that elapsed between fixation and pulling out of the junction were the measures that predicted failures in report. These findings present the possibility that these report failures could be associated with a failure in working memory, as previous research has found that working memory is increasingly important in tasks that require the integration of information over different screens [35]. These findings could therefore be important in understanding drivers’ manoeuvres at junctions which often require the integration of information over multiple fields of view while making head movements to the left and right. These findings will be discussed in more depth in regards to typical and alternative explanations for junction crashes.

## General discussion

From this series of studies, it was found that drivers failed to report approaching motorcycles on between 13% and 18% of occasions when they were pulling out in front of another vehicle. The frequency of failure to report was significantly higher for motorcycles compared to cars. These findings are a clear and dramatic demonstration of failures of situational awareness during junction crossing in a controlled environment in a high-fidelity driving simulator. Similar failures in real world junction crashes have been attributed to LBFTS errors, but the current results suggest that there may have been many occasions on which the motorcycle was actually ‘seen’ but had been subsequently forgotten before the decision to pull out was made.

There were no substantial differences in drivers’ visual attention assessed by eye movements on reported and unreported motorcycles. In contrast we did find that when drivers failed to report a motorcycle they had made more head movements and waited longer before pulling out after the initial fixation than on occasions where they reported it. Although we cannot say for certain that fixated vehicles have been processed, these findings do suggest that drivers’ fixation durations on these motorcycles were sufficiently long for them to be fully processed on other occasions. This requires us to seriously consider the possibility that on at least some occasions oncoming vehicles have been attended to and processed, but have been forgotten before the decision to pull out is made.

On trials where drivers failed to report an oncoming vehicle, our results show that there were frequent occasions where the vehicle had been fixated. Previous theories regarding attention and awareness have suggested that awareness will occur when a sufficient amount of attention is allocated to an object, therefore a longer fixation, from which we are inferring more attention, would increase the likelihood that the object will be consciously perceived [9]. If this were the case, it would have been expected that more frequent and longer fixations would be associated with reported, as opposed to unreported objects. We do not find significant evidence for this, with no large differences in the number of, or length of fixations on reported and unreported vehicles.

Of course, the fact that an object has been fixated does not guarantee that it has been encoded, change blindness and inattentional blindness experiments have demonstrated that looking at an item is not always associated with awareness [9, 10]. Participants in such experiments have frequently been shown to fail to report clearly visible objects, even on occasions when the object has been fixated. In many classic inattentional blindness experiments [36, 37], the procedure requires observers to report an unexpected, or unattended object, with the primary task being unrelated to the detection of the stimulus. The miss rates for the target item in previous paradigms has been seen to be as high as 83%^33^ and is modulated by the degree of match between the task and the items of interest [38]. However, in the current studies, the detection of approaching vehicles at the junction is the primary task for the driver, and we would therefore expect this information to be prioritised as it is critical for a safe manoeuvre.

More specifically, studies that have investigated the existence of inattentional blindness in a driving context have found that gaze durations on objects determine drivers’ ability to perceive hazards, demonstrating that eye movement dynamics can provide a measure of a driver’s hazard perception and prediction [39]. This was supported by research which investigated which eye movement measure best predicted whether a participant accurately reports an error in their own eye movements. Using a classic oculomotor capture paradigm, which encourages people to look at sudden irrelevant onsets of stimuli, it was found that longer fixation durations were associated with awareness of the error, suggesting that participants were aware of this object once it had been fixated sufficiently [22].

While the explanation of a failure in visual attention may account for at least some report failures, we must also consider the possibility that some of these errors may occur due to a failure in visual working memory. For the current findings, this explanation is particularly compelling as the results suggest that it is drivers’ subsequent behaviour which predicts their ability to report vehicles [16]. For this interpretation, information held in visual working memory may be subject to interference by subsequent visual information. Head movements in this situation will provide a large quantity of new visual information to process and retain, and there is limited reason to believe that subsequent visual behaviour after fixating on the vehicle would predict earlier attentional errors.

One might argue that if the driver had failed to encode a vehicle, and was generally expecting two vehicles in the critical memory trials, this may influence their subsequent head and eye movements when scanning the junction. If the driver had initially failed to encode a motorcycle and was expecting two vehicles then they might make additional head movements to the other side of the junction or make subsequent fixations elsewhere in the visual scene to look for another vehicle before pulling out. However, in our studies, a typical pattern of head movements involved a driver making a head movement towards a motorcyclist, a head movement to the car coming from the other direction, and a final one on the road ahead before pulling out. Inattentional blindness studies have found that the expectancy of the number of targets reduces the occurrence of miss rates in everyday tasks, as participants continue to search the scene until they have found the expected number of items [40].

The influence of traffic controls and traffic flow were not considered in the current series of studies. The current studies used a controlled intersection, with a Stop sign. Intersection crashes have been seen to be prevalent at ‘uncontrolled’ intersections [41]. Consistent with this, a previous study conducted in the same simulator has found that drivers tend to direct more attention to the side of the junction of the oncoming vehicle when there is a ‘stop sign’ compared to a ‘give way sign’ [26]. We would expect, therefore, more memory errors at uncontrolled intersections, however this is a topic for future research. In regards to traffic flow, previous research has investigated drivers’ memory for between three and eight vehicles, looking at changes in working memory load [19]. It was found that the percentage of vehicles recalled decreased with increases in memory load, with drivers on average, recalling five vehicles when there were eight vehicles present. This suggests that future work should manipulate environmental complexity, looking the links between visual scene complexity, working memory capacity and drivers’ memory for vehicles.

An additional concern with the unreported vehicles in the current studies could be that these vehicles were far away at the time of fixation and thus more likely to be unprocessed. Studies 2 and 3 were designed to ensure vehicles were presented at distances close to the gap acceptance threshold. We found that failures in reporting approaching vehicles were not related to the thresholds of individual drivers, indicating that it was not the case that unreported motorcycles were generally travelling from a further distance from the junction.

While it has been previously mentioned that studies have found drivers’ memories for their current environments to be relatively poor [19–21], explicit memory tests (where drivers are asked to recall vehicles and their locations) have been seen to not dramatically change participants’ situational awareness [42], particularly when they are actively in control of the driving task and experiencing visual-motor interactions (as here) [43]. Previous research has also found that people prefer to maintain a consistent perspective when describing spatial properties from memory [44–47]. With this in mind, our studies provide a significant advance on previous research. Firstly, our study immerses the driver in a realistic driving scenario, and finds a case where memory may nonetheless be relatively poor. Secondly, unlike previous studies our memory test was presented from the drivers’ perspective. Finally, our memory task was fully embedded, and infrequently and unpredictably presented, within a driving task. These differences make failures of memory in the current task even more surprising and encourage us to believe that they may have important implications in real driving.

These studies demonstrate that even in safety critical situations it may be possible to observe dramatic failures of visual memory–failures that could be responsible for crashes in the real world. One of the biggest challenges in this research was that such memory errors were rare—the majority of participants never made any memory errors at all. However, we were nonetheless able to observe such errors in 27 separate drivers even when they were about to pull out in front of the oncoming vehicles in Studies 2 and 3. Around 2/3 of the occasions on which these drivers pulled out in front of a vehicle that they would not subsequently remember were associated with seemingly adequate visual search. The occasional nature of such “Saw but Forgot” memory failures may be exactly why these right of way junction crashes on real roads often appear so mysterious.

**Fig 4:**
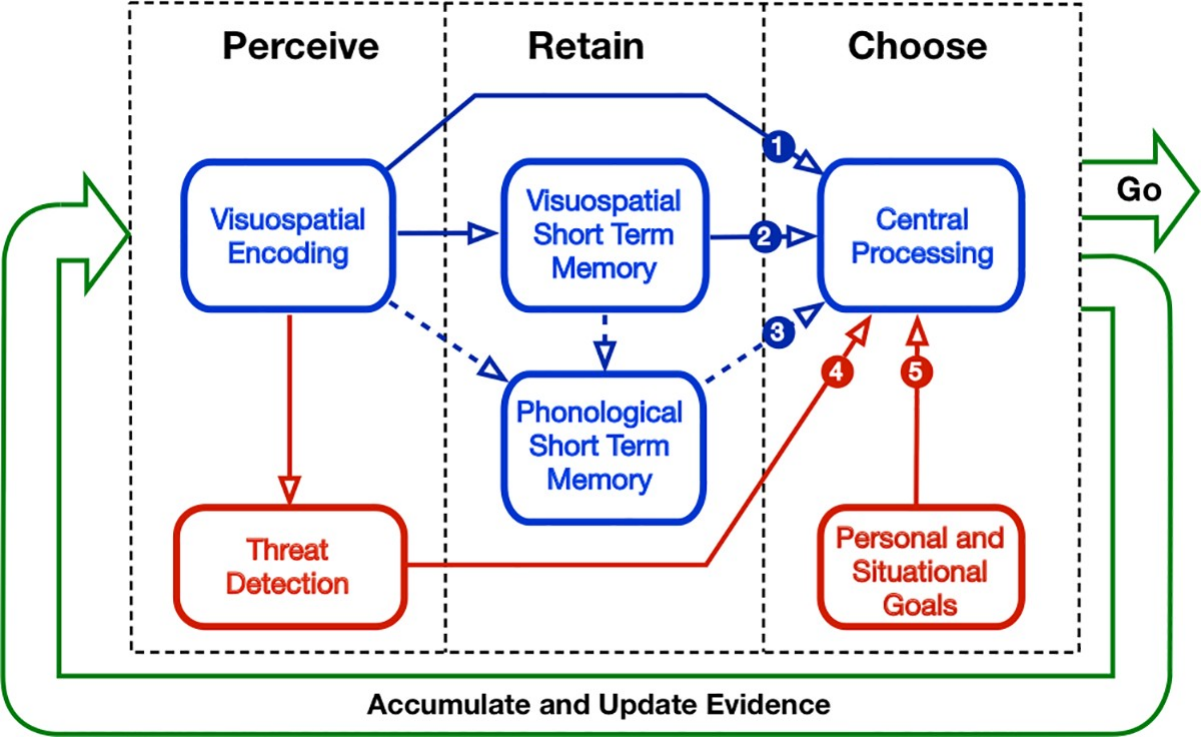
The Perceive, Retain, Choose (PRC) model has been developed as a new model of dynamic risky decision making. The role of short-term memory is explicitly emphasised, with possible processing pathways which inform a driver’s decision about when to pull out of a junction or make other risky dynamic decisions.

Traditional models [7] which have been developed to understand LBFTS errors have problems accounting for the genuine surprise frequently experienced by motorists when they have a collision of this type [7]. To help us understand how drivers could have forgotten an oncoming vehicle that they had already looked at, we have developed a new model of dynamic risky decision making in which the role of short-term memory is emphasised. The model, the Perceive, Retain, Choose (PRC) model, expands on those previously used to provide a much more explicit series of cognitive processing steps that may be involved in the decision to pull out at a junction or make other risky dynamic decisions (Fig 4).

As can be seen from Fig 4, we propose five potential pathways for relevant information to be used in the decision to pull out at a junction. Pathway 1 is the traditional account in which we look at the scene, encode the visuo-spatial information in it, and use this directly to decide whether it is safe to pull out. There are some situations where this information is all that is required, however, more commonly it will be necessary to combine visual information from one head movement with that acquired from later head movements. Pathway 2 thus involves the retention of information from the first head movement in visuo-spatial working memory. We assume that such information is retained in a limited capacity store and is available to a central processor at the same time as new information is being acquired. Clearly anything that interferes with the retention of such information will allow people to make accidental unsafe decisions. It is worth noting here that a common pattern of head movements in our studies involves a head movement towards a motorcyclist, then one to a car coming from the other direction, and a final one on the road ahead before pulling out. This raises the possibility that information from the second or third head movement has overwritten the initial contents of visuospatial memory, and that these were no longer available at the time a decision to pull out was made.

The PRC model has deliberate similarities to the predominant cognitive model of short-term memory–Alan Baddeley’s working memory model [48]. This is important as it stresses that other memory systems are potentially available. The phonological loop is a critical part of working memory theory, and suggests a simple strategy that people may be able to use in order to overcome the limited capacity of short term visuo-spatial memory. If relevant visual information is encoded phonologically, it has been shown that it is no longer subject to visuospatial interference [23]. This may be an opportunity that is underused in normal driving. In fact, people have repeatedly been shown to be unaware of the limitations in visuospatial memory [23,49], and previous research has already noted that phonological and visuospatial information may be handled differently in such situations [50]. A simple intervention that may thus prove effective to overcome interference could be teaching people that if they see a motorcycle coming, they should verbally (even sub-vocally) [51] note the fact–“See bike, say bike”. This would be represented by pathway 3 in Fig 4 and could clearly be combined with information from the earlier two pathways.

A more recent addition to Baddeley’s working memory model is the hedonic detector [52]. In the working memory model this accounts for the preferential processing of emotional information, and in the PRC model, we have included a threat detector to perform a related function. There are a number of sources of data to suggest that threatening information may be preferentially processed in this context^**8**^. Our finding that motorcyclists were systematically remembered as being further away than cars, and previous findings related to safety margin differences as a function of vehicle type [11] could all relate to the concept of threat. It is easy to understand why a visceral response to a threat may dominate any other cognitive processing [53] and pathway 4 allows for such information to have direct access to decision making. For completeness, we have also included the role of personal and situational goals in the decision. Pathway 5 allows us to understand the way in which risky decisions may differ between individuals and situations. A simple example of this is that after waiting for some time at a busy junction a driver may accept a gap size that he or she had previously rejected [54], and inter-individual variation is the motivation for the individually determined threshold procedure we have used in the current studies and elsewhere^**11**^. The PRC model is not inconsistent with the traditional account of junction crashes involving LBFTS errors, which can be represented as failures of processing leading to dangerous decisions through Pathway 1, however, it highlights a number of additional possibilities that may provide a fruitful focus for future research and practical interventions.

## Conclusions

The current studies explored drivers’ systematic biases in their memory for, perceived location of, and attention towards different vehicle types approaching an intersection in a driving simulator. Throughout this series of studies, a methodology was developed which allowed for these measures to be taken on occasions where drivers were willing to complete a manoeuvre in front of the approaching vehicles, and determine whether drivers had fixated on vehicles before subsequently failing to recall them. The most striking finding was that drivers completely failed to report some vehicles approaching the junction, particularly motorcycles, with these occasions not associated with how long the driver fixated on the vehicle for, but associated with drivers’ subsequent visual search between fixating on the oncoming vehicle and pulling out of the junction. Our results suggest that some junction crashes in which a driver reports being careful and attentive in their visual checks but nonetheless pulls out in front of an oncoming motorcycle, could be misclassified. While previous researchers suggest that this crash is associated with a failure in drivers’ visual search for motorcycles–“Look But Fail To See”, the current results highlight the possibility that at least some of these crashes could occur due to a memory deficit—a ‘Saw but Forgot’ error. These innovative and novel findings, along with the PRC model, can provide a basis for new practical interventions that may prevent SBF crashes from occurring.

## Supporting information

S1 Table A correlation of experience and the key behavioural and eye movement measures in Study 1. (PDF)

S2 Table Descriptive statistics for the Memory Test Group and Drive Only Group Comparison in Study 1. (PDF)

S3 Table Descriptive statistics for all behavioural and eye movement measures in Study 2. (PDF)

S4 Table Eye movement raw data from the 11 participants who failed to recall a motorcycle in Study 3. (PDF)

S1 File Additional statistical analysis for the factors of Vehicle Distance and Vehicle Type for the Memory Test Group and Drive Only Group comparison in Study 1. (PDF)

S2 File Study 3, between subject comparison. (PDF)

S3 File Eye movements and subsequent visual search behaviour on the unreported cars in Study 3. (PDF)
