# Supplementary material for: The ‘Saw but Forgot’ error: A role for short-term memory failures in understanding junction crashes?
Source: PLoS One. 2019 Sep 23;14(9):e0222905. doi: 10.1371/journal.pone.0222905 (PMC6756521; doi:10.1371/journal.pone.0222905)
Supplement: S1 Table — (PDF) [file pone.0222905.s001.pdf]

Correlations (n=60)		Months held licence	Annual Mileage
Mean Fixation Duration (ms)	Pearson Correlation	0.108	-0.052
	Sig.	0.413	0.694
Proportion of Fixations	Pearson Correlation	0.244	-0.052
	Sig.	0.06	0.693
Proportion of Gaze	Pearson Correlation	0.241	0.011
	Sig.	0.064	0.931
Approach Time (s)	Pearson Correlation	-0.156	-0.139
	Sig.	0.233	0.29
Cross Time (s)	Pearson Correlation	-0.083	-0.103
	Sig.	0.527	0.435
Number of Stops	Pearson Correlation	0.029	-0.122
	Sig.	0.824	0.355
Wait Time (s)	Pearson Correlation	0.121	-0.008
	Sig.	0.356	0.954
