# Supplementary material for: The ‘Saw but Forgot’ error: A role for short-term memory failures in understanding junction crashes?
Source: PLoS One. 2019 Sep 23;14(9):e0222905. doi: 10.1371/journal.pone.0222905 (PMC6756521; doi:10.1371/journal.pone.0222905)
Supplement: S2 Table — (PDF) [file pone.0222905.s002.pdf]

**Measure**	**Condition**	**Mean**	***SE***
**Behavioural Measures**			
Approach Time (s)	Memory	13.32	.35
	Drive Only	13.79	.35
Number of Stops	Memory	0.49	.07
	Drive Only	0.73	.07
Wait Time (s)	Memory	2.09	.31
	Drive Only	2.72	.31
Cross Time (s)	Memory	3.41	.14
	Drive Only	3.32	.14
**Eye Tracking Measures**			
Mean Fixation Duration (ms)	Memory	389.68*	32.69
	Drive Only	251.65*	32.69
Proportion of Fixations	Memory	.58	.03
	Drive Only	.51	.03
Proportion of Gaze	Memory	.59	.03
	Drive Only	.52	.03
