# Supplementary material for: The ‘Saw but Forgot’ error: A role for short-term memory failures in understanding junction crashes?
Source: PLoS One. 2019 Sep 23;14(9):e0222905. doi: 10.1371/journal.pone.0222905 (PMC6756521; doi:10.1371/journal.pone.0222905)
Supplement: S3 Table — (PDF) [file pone.0222905.s003.pdf]

**Measure**	**Approaching Vehicles**	**Mean**	***SE***
**Behavioural Measures**			
Thresholds (m)	Car	84.11	2.63
	Motorcycle	85.74	2.42
Estimation of Locations (degrees)	Car	21.08*	2.05
	Motorcycle	16.52*	2.10
**Eye Tracking Measures**			
Proportion of Fixations	Unreported Motorcycle	.25	.13
	Reported Motorcycle	.45	.17
Proportion of Gaze	Unreported Motorcycle	.46	.15
	Reported Motorcycle	.70	.16
Mean Fixation Duration (ms)	Unreported Motorcycle	63.76	43.12
	Reported Motorcycle	228.57	87.57
