# Supplementary material for: The ‘Saw but Forgot’ error: A role for short-term memory failures in understanding junction crashes?
Source: PLoS One. 2019 Sep 23;14(9):e0222905. doi: 10.1371/journal.pone.0222905 (PMC6756521; doi:10.1371/journal.pone.0222905)
Supplement: S4 Table — (PDF) [file pone.0222905.s004.pdf]

				Reported Motorcycle			Reported Motorcycle		
	Unreported Motorcycle			Within-Group Comparison			Between Group Comparison		
Occasion	**N of fix**	**Gaze (ms)**	**MFD (ms)**	**N of fix**	**Gaze (ms)**	**MFD (ms)**	**N of fix**	**Gaze (ms)**	**MFD (ms)**
1	2	180	90	1	60	60	2	360	180
2	1	300	300	2	120	60	2	220	110
3	1	80	80	1	80	80	1	120	120
4	1	180	180	1	360	360	2	680	340
5	1	240	240	2	120	60	2	640	320
6	1	180	180	2	600	300	1	180	180
7	1	480	480	1	120	120	1	100	100
8	1	120	120	1	80	80	1	300	300
9	1	560	560	1	200	200	1	220	220
10	1	140	140	1	300	300	1	100	100
11	1	260	260	2	400	200	1	60	60
**Mean**	**1.09**	**247.27**	**239.09**	**1.36**	**221.82**	**165.45**	**1.36**	**270.91**	**184.55**
