# Supplementary material for: The ‘Saw but Forgot’ error: A role for short-term memory failures in understanding junction crashes?
Source: PLoS One. 2019 Sep 23;14(9):e0222905. doi: 10.1371/journal.pone.0222905 (PMC6756521; doi:10.1371/journal.pone.0222905)
Supplement: S1 File — (PDF) [file pone.0222905.s005.pdf]

Drivers' Behaviour

Although it was technically possible to cross the junction in front of approaching vehicles, participants generally chose to wait for the oncoming vehicles to pass before crossing the junction so our analyses focus on the dynamics of approaching the junction, stopping at it, and then crossing. In regards to drivers' Approach Time, a main effect of Vehicle Distance was found (F (1.38, 80.02) = 43.29, MSe=12.58, *p*<.001), with contrasts revealing a linear trend (F (1, 58) = 85.13, MSe=2.94, *p*<.001). Drivers spend the shortest time in the approach zone when vehicles were at a near distance (6.4s), more time at a medium distance (8.0s) and the most time at a far distance (9.4s). There was no main effect of Vehicle Type [F (2, 116) = .99, MSe=3.70, *p*=.37].

In regards to the Number of Stops drivers made at the junction, a main effect of Vehicle Distance was found, (F (2, 116) = 17.37, MSe=.27, *p*<.001) with contrasts revealing a linear trend (F (1, 29) = 40.68, MSe=.08, *p*<.001). Drivers stopped more often when vehicles were approaching from a far distance, then a medium distance and then a near distance. There was no main effect of Vehicle Type [F (2, 116) = 2.43, MSe=.13, *p*=.10].

For drivers' Wait Time at the junction, a main effect of Vehicle Distance was found (F (1.58, 91.71) = 51.41, MSe=6.26, *p*<.001), with contrasts revealing a linear trend (F (1, 58) = 114.62, MSe=1.47, *p*<.001). Drivers stopped more often when vehicles were approaching from a far distance, then a medium distance and then a near distance. There was no main effect of Vehicle Type [F (1.86, 107.97) = .80, MSe=3.39, *p*=.45].

Finally, in regards to Cross Time, there was no main effect of Vehicle Distance [F (1.20, 69.78) = .96, MSe=1.43, *p*=.39] and no main effect of Vehicle Type [F (1.24, 71.72) = 2.31, MSe= 1.20, p=.10].

Drivers' Eye Movements

In regards to drivers' Mean Fixation Durations, there was a main effect of Vehicle Type (F (2, 116) = 12.15, MSe=54565.66, *p*<.001), with a significant contrast for motorcycles vs. cars and large vehicles: F (1, 58) = 6.33, MSe=508284.776, *p*<. 05). Drivers had lower mean fixation durations to the side of the junction when a motorcycle was present compared to a car or large vehicle. There was no main effect of Vehicle Distance [F (2, 116) = 2.44, MSe=55.484.38, *p*=.06].

In regards to Proportion of Fixations, there was no main effect of Vehicle Type [F (2, 116) =2.30, MSe=.06, *p*=.11] and no main effect of Vehicle Distance [F (2, 116) =.19, MSe=.08, *p*=.83]. For Proportion of Gaze, there was also no main effect of Vehicle Type [F (2, 116) = 3.00, MSe=.07, *p*=.06) and no main effect of Vehicle Distance [F (2, 116) = .33, MSe=.08, *p*=.72].
