# Supplementary material for: The ‘Saw but Forgot’ error: A role for short-term memory failures in understanding junction crashes?
Source: PLoS One. 2019 Sep 23;14(9):e0222905. doi: 10.1371/journal.pone.0222905 (PMC6756521; doi:10.1371/journal.pone.0222905)
Supplement: S2 File — (PDF) [file pone.0222905.s006.pdf]

Eye movement measures were compared between the trials where the motorcycle was not reported and matched trials where another participant reported the motorcycle. Another 11 participants who reported the motorcycle on the right-hand side of the junction were matched on the basis of combined gap acceptance thresholds.

Again, it was found that there was no difference in the number of fixations [$t(20) = 1.54$, $p=.14$], total gaze duration [$t(20) = .30$, $p=.77$] and mean fixation durations [$t(20) = .98$, $p=.34$] on unreported and reported approaching motorcycles. See s6 for the full eye movement descriptive statistics for the 11 participants who failed to recall a motorcycle, their reported within group comparison, and the reported between subject comparison.
