# Supplementary material for: The ‘Saw but Forgot’ error: A role for short-term memory failures in understanding junction crashes?
Source: PLoS One. 2019 Sep 23;14(9):e0222905. doi: 10.1371/journal.pone.0222905 (PMC6756521; doi:10.1371/journal.pone.0222905)
Supplement: S3 File — (PDF) [file pone.0222905.s007.pdf]

Out of the 3 occasions were drivers failed to recall a car, on 1 of these occasions the driver fixated on the approaching car however, on 2 of the occasions the driver did not fixate on the approaching car.
Eye movement measures were compared between the trials where the driver failed to report and successfully reported the car. The number of fixations on the oncoming car was identical for the unreported trial and reported trial, with 1 fixation. The total gaze duration on the unreported trial was 264ms and on the reported trial was 260ms.

In terms of the subsequent eye movements, the driver made 2 subsequent fixations on the unreported and reported trial, made 1 subsequent head movement on the unreported and reported trial however, the time of the last fixation on the car before pulling out of the junction was earlier for the unreported car (1900 ms) compared to the reported car (1460 ms). Further investigations are needed to confirm whether the differences in eye movements between the unreported and reported motorcycles (see main article) are specific to motorcycles.
